# Study protocol for a non-inferiority trial of cytisine versus nicotine replacement therapy in people motivated to stop smoking

**DOI:** 10.1186/1471-2458-11-880

**Published:** 2011-11-21

**Authors:** Natalie Walker, Colin Howe, Chris Bullen, Hayden McRobbie, Marewa Glover, Varsha Parag, Jonathan Williman, Reon Veale, Vili Nosa, Joanne Barnes

**Affiliations:** 1 Clinical Trials Research Unit, School of Population Health, The University of Auckland, Private Bag 92019, Auckland 1142, New Zealand; 2 UK Centre for Tobacco Control Studies, Wolfson Institute of Preventive Medicine, Barts and the London School of Medicine and Dentistry, Queen Mary University of London, UK; 3 Centre for Tobacco Control Research, Social and Community Health, School of Population Health, The University of Auckland, Private Bag 92019, Auckland 1142, New Zealand; 4 The Quit Group, PO Box 12605, Wellington, New Zealand; 5 Pacific Health, School of Population Health, The University of Auckland, Private Bag 92019, Auckland 1142, New Zealand; 6 School of Pharmacy, The University of Auckland, Private Bag 92019, Auckland 1142, New Zealand

## Abstract

**Background:**

Smokers need effective support to maximise the chances of successful quit attempts. Current smoking cessation medications, such as nicotine replacement therapy (NRT), bupropion, nortriptyline or varenicline, have been shown to be effective in clinical trials but are underused by smokers attempting to quit due to adverse effects, contraindications, low acceptability and/or high cost. Cytisine is a low-cost, plant-based alkaloid that has been sold as a smoking cessation aid in Eastern Europe for 50 years. A systematic review of trial evidence suggests that cytisine has a positive impact on both short- and long-term abstinence rates compared to placebo. However, the quality of the evidence is poor and insufficient for licensing purposes in many Western countries. A large, well-conducted placebo-controlled trial (n = 740) of cytisine for smoking cessation has recently been published and confirms the findings of earlier studies, with 12-month continuous abstinence rates of 8.4% in the cytisine group compared to 2.4% in the placebo group (Relative risk = 3.4, 95% confidence intervals 1.7-7.1). No research has yet been undertaken to determine the effectiveness of cytisine relative to that of NRT.

**Methods/design:**

A single-blind, randomised controlled, non-inferiority trial has been designed to determine whether cytisine is at least as effective as NRT in assisting smokers to remain abstinent for at least one month. Participants (n = 1,310) will be recruited through the national telephone-based Quitline service in New Zealand and randomised to receive a standard 25-day course of cytisine tablets (Tabex^®^) or usual care (eight weeks of NRT patch and/or gum or lozenge). Participants in both study arms will also receive a behavioural support programme comprising an average of three follow-up telephone calls delivered over an eight-week period by Quitline. The primary outcome is continuous abstinence from smoking at one month, defined as not smoking more than five cigarettes since quit date. Outcome data will also be collected at one week, two months and six months post-quit date.

**Discussion:**

Cytisine appears to be effective compared with placebo, and given its (current) relative low cost may be an acceptable smoking cessation treatment for smokers, particularly those in low- and middle-income countries. Cytisine's 'natural' product status may also increase its acceptability and use among certain groups of smokers, such as indigenous people, smokers in countries where the use of natural medicines is widespread (e.g. China, India), and in those people who do not want to use NRT or anti-depressants to help them quit smoking. However it is important to ascertain the effectiveness of cytisine compared with that of existing cessation treatments.

**Trial registration:**

Australian New Zealand Clinical Trials Registry (ACTRN12610000590066)

## Background

One in two smokers will die of a smoking-related disease, and half of those who die as a direct consequence of smoking will die in middle age [1]. Smoking (combining active and passive smoking) is responsible for approximately 18% of all deaths in New Zealand [2], an island nation (population 4.3 million) in the Southwest Pacific. Smoking contributes substantially to the life expectancy differential between Māori (indigenous New Zealanders who comprise 15% of the national population) and non-Māori [3]. Stopping smoking at any age reduces smoking related risks and increases life expectancy [4]. Effective treatments to aid smoking cessation have clear individual and public health benefits and provide the most efficient use of health care resources at a population level [5].

Use of pharmacological treatments such as nicotine replacement therapy (NRT), bupropion and nortriptyline approximately doubles a smoker's chance of long-term abstinence [6,7]. Varenicline appears superior to bupropion and at least doubles the long-term chances of quitting compared to placebo [8]. The success of a smoking cessation product depends on its availability and acceptability to smokers as well as its efficacy. In 2009 in New Zealand, among smokers aged 15-64 years who had made a recent quit attempt, 22% had used NRT and 4.6% had used a prescription medicine (e.g. varenicline) to support their quit attempts [9]. Many smokers are misinformed about the risks of nicotine and are therefore hesitant to use NRT [10,11]. Bupropion and nortriptyline have a number of adverse effects and contraindications [7], which may contribute to their relatively poor uptake among smokers trying to quit. Given these issues new pharmacological treatments to help people quit smoking need to be identified - one such product is cytisine.

Cytisine is an alkaloid found in plants such as the Golden Rain tree (*Cytisus laburnum*) [12] and the New Zealand Kowhai tree (*Sophora tetraptera*) [13]. Such plants are considered to be poisonous to humans, with the alkaloids they contain (including cytisine) considered to be the toxic components. However, an oral tablet form of cytisine, Tabex^®^, has been marketed by Sopharma (Bulgaria) as a smoking cessation aid since the 1960's. When used at its therapeutic dose (1.5-9 mg of cytisine per day) trial data indicates that Tabex^® ^is well tolerated with few adverse effects, similar to those seen with NRT use [14-17]. Furthermore, West et al. (2011) report that a recent Periodic Safety Update provided to European authorities, did not identify any safety signals for cytisine (based on a sample of more than 7 million exposed persons) [17]. Cytisine overdose has been reported and is likened to nicotine intoxication, with symptoms including nausea, vomiting, pupil dilation, tachycardia, general weakness, clonic convulsions, and respiratory paralysis [18]. Cytisine is off-patent and currently relatively inexpensive compared with most other pharmacological cessation products on the market. Cytisine may also be attractive to those people who prefer not to use a nicotine-based product or antidepressants to help them quit smoking. A recent qualitative study looking at cytisine's potential to be used as a healing remedy by traditional indigenous healers to help their people stop smoking, suggests that cytisine would be very attractive if it were marketed in a culturally appropriately manner [19].

Like its analogue varenicline, cytisine acts as a partial agonist of the nicotinic acetylcholine receptor [8]. It is thought to aid smoking cessation by reducing the reward and satisfaction associated with smoking [20]. Despite having been used by hundreds of thousands of smokers throughout Eastern Europe [20], cytisine is rarely mentioned in English-language literature and there are only limited data from clinical trials to support its use. A systematic review [14] identified three placebo-controlled trials of cytisine and all were published in non-English language journals. All three trials were conducted over 30 years ago in Eastern Europe prior to the advent of good clinical practice (GCP) guidelines and all suffered from weaknesses in study design and reporting of results [21-23]. The authors of the review combined data from these trials in a meta-analysis and reported a clear effect on smoking abstinence at 3-8 weeks (Odds Ratio [OR] 1.93, 95% confidence interval [CI] 1.21 to 3.06) and at 3-6 months (OR 1.83, 95% CI: 1.21 to 2.99) for cytisine compared to placebo [14]. Although these findings appear promising, the three trials were not conducted to GCP standards and thus regulatory authorities in many countries are unlikely to accept the evidence for licensing purposes. Data from at least two appropriately powered placebo-controlled clinical trials run to GCP standards are required to satisfy regulatory authorities in most western countries. Since the review was published, two additional placebo-controlled trials of cytisine have been published and both confirm earlier findings [16,17]. The first trial was well-conducted, with randomisation, double-blinding and clear outcome measures, but was underpowered (n = 171). The authors reported an OR for cessation at 26 weeks of 8.93 (95% CI: 1.06 to 75.28). A second trial had a large sample size (n = 740), was conducted to GCP standards and reported 12-month continuous abstinence rates of 8.4% in the cytisine group compared to 2.4% in the placebo group (relative risk=[RR] 3.4, 95% CI 1.7-7.1) [17].

Following on from the recently published placebo-controlled trial by West et al. (2011) [17], an appropriate research question to now answer is how the efficacy of cytisine compares to NRT. Such a question is important from a policy and funding perspective to help decide whether to use cytisine as a first or second line agent for smoking cessation treatment or whether to publicly fund the drug. Based on the systematic review mentioned above, the expected difference in efficacy between cytisine (OR = 1.83, 95% CI 1.21 to 2.99)[14] and NRT (OR = 1.74, 95% CI 1.64 to 1.86) [6] is too narrow to feasibly conduct a head-to-head superiority trial. We therefore propose conducting a non-inferiority trial to demonstrate whether cytisine is at least as effective as NRT at increasing quit rates in dependent smokers motivated to quit.

### Objectives

The primary objective of the trial is to determine how effective cytisine is at increasing quit rates at four weeks after randomisation. This study has three main hypotheses: 1) that cytisine plus behavioural support is at least as effective as usual care (NRT plus behavioural support) at increasing quit rates; 2) that cytisine plus behavioural support is at least as effective as usual care (NRT plus behavioural support) at reducing the severity of withdrawal symptoms; and 3) that cytisine is an acceptable smoking cessation treatment to Māori, Pacific and non-Māori non Pacific alike.

## Methods/Design

### Study design

This study is a parallel group, single-blind, non-inferiority, randomised controlled clinical trial.

### Study population

The study population will be registered and eligible callers to the national toll-free smoking cessation helpline [Quitline] in New Zealand.

### Inclusion criteria

Participants will be smokers from throughout New Zealand who want to stop smoking, are at least 18 years of age, are able to provide verbal consent, and have access to a telephone.

### Exclusion criteria

Pregnant women and women who are breastfeeding will be excluded from the trial. People will also be excluded from the trial if they meet any of the following criteria: current users of pharmacological cessation therapies (e.g. NRT, buproprion, clonidine, nortriptyline or varenicline); current clients of another smoking cessation programme or cessation study; they have uncontrolled high blood pressure (> 150 mmHg systolic, > 100 mmHg diastolic), schizophrenia or phaeochromocytoma; they have had a myocardial infarction, angina, severe cardiac arrhythmia or a stroke in acute phase within the last two weeks. The medical conditions above are listed contraindications for the use of cytisine, NRT patch, gum, and lozenge. A number of disease conditions are known where precautions for the use of NRT products are indicated, and will be assessed in each participant by researchers prior to randomisation. These include: severe cardiovascular disease (occlusive peripheral arterial disease, cerebrovascular disease, stable angina pectoris, and uncompensated heart failure) and vasospasm, uncontrolled hypertension, renal or hepatic impairment, active duodenal ulcers and gastric ulcers.

### Randomisation: allocation concealment and sequence generation

All participants will be assigned a unique registration number allocated by a central computer following details submitted on a web-based form. This number will be used to identify each randomised participant who has consented to take part. Participants will be randomised by computer, with stratified minimisation by sex, ethnicity (Māori, Pacific, non-Māori non-Pacific), and level of nicotine dependence (> 5 points, ≤ 5 points on the Fagerström Test for Nicotine Dependence [FTND] questionnaire [24]), to ensure a balance in these key prognostic indicators between the intervention and control groups. The randomisation sequence will be concealed from all research staff and will not be able to be manipulated.

### Blinding

Due to the nature of the intervention only single blinding (of researchers, not of participants) is possible. Members of the trial steering committee, management committee, and other study team members (with the exception of the project co-ordinator and the Quitline senior research manager) will remain blinded to treatment allocation until the code is broken (namely, after the last follow-up call is completed and the data recorded). The project co-ordinator will not be blinded as they will be responsible for distributing the cytisine to participants. The senior research manager and research assistants based at Quitline will not be blinded to treatment allocation, as they are required to know which group each participant has been allocated to in order to conduct the scheduled follow-up calls.

### Study intervention

**Figure 1:**
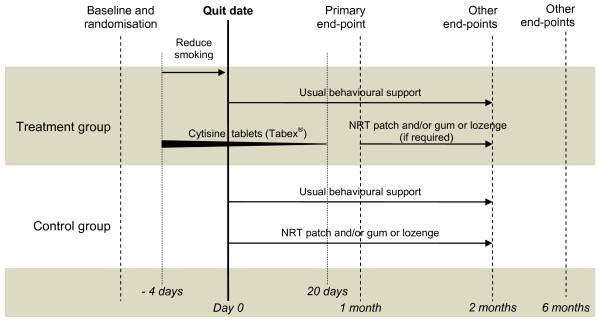
**Trial schematic**. Footnote: NRT = Nicotine replacement therapy.

Participants will be randomised to receive a course of cytisine [intervention] or usual care [control] (Figure 1). All participants will also receive an eight week, telephone-based, behavioural support programme delivered by Quitline advisors. Quitting support generally involves an average of three follow-up telephone calls, each lasting about 10-15 minutes. However, if people do not want to receive this support, they are not scheduled and callers are advised that they can phone Quitline at any time for support.

#### Intervention

Participants in the intervention arm will receive a 25-day course of cytisine tablets (Tabex^®^) delivered by courier. Participants will be asked to reduce their smoking over the first four days of treatment so that they are not smoking at all by the fifth day, which will be their designated Quit date. Participants will follow the dosing regimen as recommended by the manufacturer, namely:

• Days 1-3: 1 tablet every 2 hours through the waking day (up to six tablets per day)

• Days 4-12: 1 tablet every 2.5 hours (up to 5 per day); designated Quit date is day 5

• Days 13-16: 1 tablet every 3 hours (up to 4 per day)

• Days 17-20: 1 tablet every 4-5 hours (3 per day)

• Days 21-25: 1 tablet every 6 hours (2 per day)

As part of an automated process at Quitline, all participants, including those in the treatment group, will be sent Quitcards, which may be redeemed for subsidised NRT (see below for more detail). However, participants will be asked to use the cytisine tablets sent to them for the first 25 days, and not the NRT. Participants will be told to redeem their vouchers for NRT only if they are smoking one month after their trial quit date or require on-going cessation support after they have finished the course of cytisine.

#### Control

Participants allocated to the control arm of the trial will be asked to stop smoking completely on their chosen Quit date and will be supported by Quitline in the usual way for eight weeks, with counselling/advice and NRT in the form of a patch and/or gum or lozenge. Current standard cessation practice at the New Zealand Quitline (as at November 2011) is as follows (other cessation providers in New Zealand do not follow the process outlined here): Quitline issues by post, two (or sometimes three) four-week Quitcards (with a 90 day expiry). Participants take each four-week Quitcard to a pharmacist in exchange for subsidised patches and/or gum or lozenges (NZ$3-6 per item per four-week course of NRT). The strength of NRT patch and/or gum or lozenges to be used by each participant is determined by the Quit advisor (as per their dosage guidelines) as to the degree of each person's nicotine dependency.

### Baseline assessments

Participants from throughout New Zealand will call Quitline seeking advice and wishing to enrol in the Quitline smoking cessation programme. A Quit advisor from Quitline will undertake the standard Quitline assessment. A list of those individuals who indicate "yes" to taking part in research will be transferred from the Quitline database to the research assistants based at Quitline. The research assistants will attempt to make contact with people on the list. Those contacted will be told about the trial and will be asked if they would like to take part in the study. If the potential participant indicates that they are interested, they will be consented and asked for demographic data (age and sex) and checked to see if they meet the inclusion criteria for the trial. For those who do meet the inclusion criteria, further details will be collected about the participant's ethnicity and level of nicotine dependence (as determined by the FTND [24]) and they will be randomised. Data from this initial assessment will then be electronically transferred (securely) to the Clinical Trial Research Unit's Oracle database. The research assistant will then collect the following baseline data from each participant over the telephone.

• *Demographic information: *Socio-economic position based on education.

• *Smoking history: *Age when started, number of cigarettes smoked per day, number of years as regular smoker, number of previous unsuccessful attempts to give up in past 12 months and the method used, type of cigarettes smoked per day (e.g. roll-your-own or factory-made) and usual brand/pack size (and how long pack lasts for roll-your-own users).

• *Other smoking related information: *Self-rated chances of quitting, household smoking, and smoking in cars. Satisfaction with smoking will also be assessed at baseline (and at follow-up), using the modified Cigarette Evaluation Questionnaire (mCEQ) [25]. This questionnaire was used in the one trial (n = 746) that has compared the effect of varenicline versus NRT on quit rates [26]. This trial found varenicline use resulted in significantly less satisfaction with smoking compared to NRT use, in those participants that did not quit smoking. It is important to determine if cytisine, given it is a similar class of drug to varenicline, has an analogous effect.

• *Concomitant medication: *Information about types of medication currently used will be collected.

• *The physical signs and symptoms associated with withdrawal: *Measured using the Mood and Physical Symptoms Scale (MPSS) [27].

• *Alcohol use and abuse: *Data from both animal [28] and human studies [29] have shown that varenicline can also reduce alcohol consumption. Given cytisine is a similar class of drug as varenicline it may have an analogous effect on alcohol use, although to date no such effect has been published. For this reason, information about alcohol use and abuse will be collected in this study, using the Alcohol Use Disorders Identification Test-consumption subscale [AUDIT-C], a screening tool that helps to identify people who are hazardous drinkers or have an active alcohol use disorder (including alcohol abuse or dependence) [30].

### Outcome measures

#### Primary outcome

The proportion of participants that have stopped smoking, one month post-quit. 'Stopped smoking' will be defined as self-report of having smoked not more than five cigarettes in the one month after the Quit date (continuous abstinence).

#### Secondary outcome

The following secondary outcome measures will be assessed at one week and one month after the Quit date in the treatment group only:

• *Information related to the use of cytisine: *Participants will be asked for their views on the use of cytisine as a cessation aid (i.e. whether they would recommend the treatment to another smoker and whether they liked or disliked using the product).

• *Participants' treatment compliance: *Self-reported pill counts (also asked at two months), concomitant cytisine and NRT use (also asked at two months), early stopping of cytisine and reasons why.

The following secondary outcome measures will be assessed in all participants at one week and one, two and six months after the Quit date:

• *The physical signs and symptoms associated with withdrawal: *Assessed using the MPSS [27] in abstinent smokers, measured at one week, one month and two months only.

• *Self-rated chances of quitting: *Measured at one week, one month and two months only.

• *Seven-day point prevalence: *The proportion of participants that have stopped smoking, defined as self-report of having smoked no cigarettes (not even a puff) in the past seven days. Measured at every time point.

• *Continuous abstinence: *The proportion of participants that have stopped smoking, defined as self-report of smoking not more than five cigarettes from the Quit date. Assessed at every time point.

• *Use of cigarettes: *If the participant is smoking at any time point, the following outcomes will be assessed: number of cigarettes smoked per day; proportion of participants who have significantly reduced their daily smoking level (defined as reducing consumption by at least 25% in terms of numbers of cigarettes per day or weight of loose tobacco per day); smoking satisfaction (measured using the mCEQ).

• *Use of NRT: *All participants will be asked about use of any NRT (including type, dosage, flavour, and frequency of use) at every time point.

• *Use of non-NRT methods of cessation: *Participants will be asked about their use of non-NRT methods of cessation such as buproprion, clonidine, nortriptyline, varenicline, acupuncture etc, measured at two and six months only.

• *Alcohol use and abuse: *Measured using the AUDIT-C. Measured at one and two months only.

• *Adverse events: *Information regarding any adverse events and whether they are considered to be related to treatment will be collected at every time point.

• *Cost information: *Cost outcomes will be derived from known costs of the various products, and will include cost per quitter and cost per person reducing their daily cigarette consumption. The tobacco expenditure savings to individual smokers will also be calculated using data on the daily amount smoked prior to quitting and the price of the particular products smoked.

• *Concomitant medication: *Collected at every time point.

### Sample size

A sample size of 1,310 (655 in each group) is required for this trial. Internal Quitline service evaluations and previous findings from a cessation trial conducted by the Clinical Trials Research Unit have established that the average four week quit rate in people receiving usual care through Quitline is approximately 50% [31,32]. A non-inferiority margin of difference between the group proportions was set at 5% (i.e. the quit rates in the cytisine treatment group will be no worse than 5% less than the rate in the NRT active control group). A sample size of 1,310 will be sufficient to confer 90% power at the one-sided significance level of 0.025 using a binomial test for equivalence. The true four week quit rate in those who receive cytisine was assumed to be 55%, which is midway between the estimated efficacy of varenicline (60%) [8] and NRT [6]. The required sample size incorporates a 20% inflation factor to allow for a sufficiently large dataset to evaluate the intervention based on both the intention to treat as well as the per protocol population. The sample size is adequately powered to also evaluate the non-inferiority of cytisine on two month quit rates. Recruitment will take approximately 18 months based on previously run Quitline trials [32,33], and will aim for a minimum of 25% of participants to be Māori and 15% to be Pacific peoples. The sample size will allow the consistency of effects for Māori and Pacific peoples compared to non-Māori non-Pacific to be assessed.

### Withdrawal criteria

Should participants require discontinuation of study treatment for any reason (see below), or if they elect to cease taking treatment, follow-up calls and data collection will continue as scheduled as if they were continuing with the randomised treatment. Participants may have the study treatment withdrawn if one or more of the following occurs:

• The participant makes a voluntary decision to withdraw from follow-up, or from the treatment.

• The participant has any serious clinical adverse event, concurrent illness, or other medical condition that indicates to the principal investigator that continued treatment with the study treatment is not in the best interest of the participant. The study treatment will be withdrawn if the participant develops any life threatening or seriously disabling illness or is admitted to hospital.

• The participant becomes pregnant during the course of the trial. Participants will be advised to stop taking their allocated treatment and discuss on-going use of smoking cessation products with their general practitioner or lead maternity caregiver.

• The principal investigator feels that it is in the best interests of the participant.

• The study is terminated.

If the participant discontinues treatment due to a serious adverse event, the participant will be followed until the event resolves or there is a return to a clinically acceptable medical status. Participant deaths or serious adverse events, which occur within 30 days of discontinuation, will be reported to the project co-ordinator.

### Data management

The design and management of all databases associated with this trial will be undertaken by the data management and information technology groups at the Clinical Trials Research Unit. The databases will be constructed in Oracle and internet database entry at Quitline (undertaken by the research assistants) will be via their in-house Filemaster system, from which the Unit receives hourly updates. Uploads will be stored in the Unit's Oracle database and linked to the electronic Case Record Form (CRF) data where applicable. Validation rules for each CRF will be specified by the project co-ordinator, in association with the senior data manager. These rules will include range checks so that inaccuracies in data collection can be identified early. A query will be raised as soon as any values are entered that are outside the allowed range or if data are missing. The research assistants at Quitline will amend the form as soon as a query is raised.

### Data monitoring

An independent Data Safety and Monitoring Committee (DSMC) will be established for this trial. Members of the committee will have no conflicts of interest and will not be directly involved with the trial. The DSMC will draw up their own terms of reference and will be free to review any information or study process in addition to the reviews of safety data. The study statistician will provide the committee with reports on safety data. An independent person will also monitor Quitline and the Clinical Trials Research Unit during the trial to ensure that the study protocol is adhered to. At Quitline the monitor will audit every randomised participant's records and ensure that study documentation is up-to-date and record keeping meets protocol and regulatory requirements. The monitor will visit Quitline early on during the study (after ten participants have been randomised), at study close-out and twice during the course of the trial. The monitor will audit the Clinical Trials Research Unit every three months to see that handling of the study medication is appropriate and correct documentation is kept.

### Data analysis

A senior biostatistician at the Clinical Trials Research Unit was involved in the sample size estimation, wrote the statistical analysis plan agreed upon by all members of the Steering Committee specifying *a priori *all analyses to be undertaken. Data from the trial will be entered into an Oracle database and all statistical analyses will be performed using SAS version 9.2 (SAS Institute Inc. Cary NC), and R. No interim analyses will be undertaken.

#### Baseline characteristics

Socio-economic status, smoking information, and mood and physical symptoms associated with withdrawal will be summarised and descriptive summary statistics provided. Since any differences between randomised groups at baseline could only have occurred by chance, no formal significance testing will be conducted.

#### Treatment effects

Non-inferiority will be evaluated by testing whether the lower bound of the 95% CI for difference in quit rates excludes a 5% difference. In particular, the proportion of participants that have been continuously abstinent at four weeks for both groups and the associated two-sided 95% CI for the difference will be estimated. Non-inferiority of cytisine over NRT will be accepted if the lower bound of the 95% CI around the estimated difference in the primary endpoint event rates lies above 5%. The primary analyses will be carried out on an intention-to-treat basis where participants lost to follow-up will be presumed to be smoking. However, it is increasingly recognised that non-inferiority studies should also be evaluated against a per protocol population defined on the basis of compliance, protocol violations, and missing data [34,35]. Both sets of results are important and will be considered when assessing the study objective. In the case that non-inferiority is evident, assessment as to whether cytisine is superior to NRT will be carried out using the same approach but comparing to a zero difference and applied to the intention to treat population.

Chi-squared analyses comparing the proportion of participants that have been continuously abstinent by treatment group, and incidence rates, RR, risk differences and 95% CI will be calculated. Subsequent secondary analyses involving adjustment for baseline covariates including stratification factors will be undertaken using logistic regression. The consistency of effects for Māori, Pacific peoples, and non-Māori non-Pacific will be assessed using tests for heterogeneity. The distribution of all continuous endpoints will be assessed for normality and skewed data will be subjected to an appropriate transformation before analysis. The change from baseline in symptoms of nicotine withdrawal, AUDIT-C and number of cigarettes smoked per day will be analysed using repeated measures models and adjustment for baseline value.

A pre-analysis review will be conducted before the primary analyses. The purpose of this review is to establish rules for a per protocol analysis and to reconsider the analysis plan (e.g., adjustment for potential confounders) in light of recent research. Any amendments (and the rationale) will be justified and documented in the statistical analysis plan. A per protocol analysis involves looking at the major protocol violations such as cross-over treatments, withdrawals and lost to follow-up. The per protocol population will consist of all randomized patients who have complied with the treatment allocated, were not lost to follow-up, and who do not have major protocol deviations pertaining to eligibility or the assessment of the treatment difference.

#### Cost analyses

If the primary outcome of the trial is positive then cost analyses will be undertaken. Cost outcomes will include cost per quitter and cost per person reducing their daily cigarette consumption. These data will then be compared with New Zealand data from Quitline and other NRT service providers, in addition to information from various international studies. This modelling will take a health sector perspective. However, the tobacco expenditure savings to individual smokers will also be calculated (for those who quit and cut down) to give a more societal perspective on the benefits (especially to low-income smokers). This calculation will use data on the daily amount smoked prior to quitting and the price of the particular products smoked. For those who cut down their consumption by a significant margin (i.e. 25% or more), the cost per person reducing their daily cigarette consumption will be calculated.

#### Tolerability

All randomised participants who receive at least one dose of intervention or control treatment will be included in the planned analyses. Comparison of the frequency of treatment withdrawal between the intervention and the control group will be tested using Chi-square statistics. The numbers of participants discontinuing treatment prematurely for any reason will be summarised by treatment group and by reasons for discontinuation. The incidence of all suspected serious adverse treatment reactions will be summarised by treatment group.

### Ethics

Ethics approval has been granted by the National Multi-Regional Ethics Committee (Ethics number MEC/10/08/078). Maintenance of confidentiality and compliance with the Privacy Act will be emphasised to all study participants. Participation in the study is entirely voluntary. Verbal consent will be obtained at the time of contact with Quitline, however a written consent form and patient information sheet will be posted out to participants for their information. Data will be entered, stored and backed-up in a secure manner via the Unit's internet data management system. Participants will be acknowledged in all publications and presentation of the results, and will receive a copy of the trial findings written in lay terms.

## Discussion

### Primary outcome assessment

The non-inferiority trial design precludes us from having a primary outcome based on six month quit rates. Loss to follow up and treatment non-compliance tends to reduce observed differences in treatment effect, which in non-inferiority trials biases findings away from the null. Later assessments are more likely to suffer from loss to follow up and treatment non-compliance, particularly as participants in the intervention group may use NRT after they have completed their 28 day course of cytisine if they feel it necessary to support their on-going quit attempt. Removing these participants from per protocol analysis of six month quit rates is likely to significantly reduce the power of the study. Instead the primary outcome will be based on a one month assessment of quit rates, with six month quitting variables collected as secondary outcomes.

### Validation of smoking abstinence

No validation of self-reported smoking status will be undertaken in this trial. Study participants will be located throughout New Zealand (a country of approximately 1,600 km in length) and for budgetary and logistical reasons it is not possible to conduct face-to-face verification of their smoking status. Furthermore, biochemical tests which measure cotinine, a metabolite of nicotine, cannot be used to validate the primary endpoint as a large proportion of control participants will be using NRT at this time. The Society for Research on Nicotine and Tobacco reports that the validity of self-reported smoking status is consistently high in large population-based studies and that attempts to validate quit status may introduce a selection bias unrelated to participants' smoking status [36].

### Current status

The trial started recruiting on the 1st April 2011, with recruitment expected to take 18 months. Trial findings are likely to be available late 2013.

## List of abbreviations

AUDIT-C: Alcohol Use Disorders Identification Test-consumption subscale; CI: Confidence Interval; CRF: Case Record Form; DSMC: Data Safety Monitoring Committee; FTND: Fagerström Test for Nicotine Dependence; GCP: Good Clinical Practice; mCEQ: modified Cigarette Evaluation Questionnaire; MPSS: Mood and Physical Symptom Scale; NRT: Nicotine Replacement Therapy; OR: Odds Ratio; RR: Relative Risk; SAS: Statistical Analysis Software.

## Competing interests

We, as the authors of this article have no competing interests (financial or otherwise) in this publication. The Tabex cytisine tablets used in this trial have been provided by Sopharma, Bulgaria. NRT patches, gum and/or lozenges used by all study participants after Quit day are purchased by participants (using the subsidised Quitcards they receive from Quitline) from community pharmacies. No authors have received support from any companies for the submitted work. No authors have any relationship with Sopharma, a company that might have an interest in the submitted work. HM has received honoraria for speaking at research symposia and received benefits in kind and travel support from, and has provided consultancy to the manufacturers of smoking cessation medications. CB and HM have previously undertaken research on behalf of NicoNovum (a manufacturer of smoking cessation medications), but prior to the purchase of the company by RJ Reynolds. NW has provided consultancy to the manufacturers of smoking cessation medications, received honoraria for speaking at a research meeting and received benefits in kind and travel support from a manufacturer of smoking cessation medications. MG and JW have provided consultancy to the manufacturers of smoking cessation medications. JB was previously (1999-2002) a Lichtwer research fellow, has undertaken research funded by and has received benefits in kind and travel support from LichtwerPharma (a manufacturer of a herbal medicine used in smoking cessation). The author's spouses, partners, or children have no financial relationships that may be relevant to the submitted work. All authors have no non-financial interests that may be relevant to the submitted work.

## Authors' contributions

NW, JB, CB, MG, HM, VN, and JW conceived the original idea for the trial, sought funding and wrote the protocol. CH and RV manage the day to day running of the trial, including all participant follow-up. VP will undertake all data analyses. This protocol paper was written by NW and JW, with input from all co-authors. NW will act as guarantor for this paper. All authors have read and approved the final manuscript.

## Pre-publication history

The pre-publication history for this paper can be accessed here:

http://www.biomedcentral.com/1471-2458/11/880/prepub
